# Glucose–TOR Signaling Regulates Root Hair Elongation in *Arabidopsis* via the RHD6-RSL4 Transcriptional Cascade

**DOI:** 10.3390/plants15172586

**Published:** 2026-08-25

**Authors:** Bingru Wang, Jueru Zhang, Wei Yan, Xiumei Dai, Jiankui Zhang, Tian Zhang, Kexuan Deng

**Affiliations:** 1 College of Agronomy and Biotechnology, Southwest University, Chongqing 400715, China; 18042635531@163.com (B.W.); z02857512@163.com (J.Z.); 19848985054@163.com (W.Y.); dxm72031@swu.edu.cn (X.D.); jkzhang@swu.edu.cn (J.Z.); 2 Engineering Research Center of South Upland Agriculture, Ministry of Education, Chongqing 400715, China; 3 Chongqing Medical and Pharmaceutical College, Chongqing 401331, China

**Keywords:** TOR, root hair elongation, RHD6-RSL4 cascade, RHS genes

## Abstract

Root hairs are tubular protrusions of root epidermal cells that expand the root surface area to facilitate water and nutrient uptake. The target of rapamycin (TOR) kinase has been identified as a positive regulator of root hair elongation, and the RHD6-RSL4 bHLH transcriptional cascade is well established as a core module that governs root hair morphogenesis. However, whether TOR signaling acts upstream of the RHD6-RSL4 pathway and how glucose signals are integrated into this transcriptional regulatory network during root hair development remain incompletely understood. In this study, transcriptome profiling combined with pharmacological and genetic functional assays was performed to elucidate the TOR-mediated transcriptional regulatory pathway of root hair elongation in *Arabidopsis*. Chemical inhibition of TOR triggered genome-wide transcriptional reprogramming in seedling roots, including disruption of auxin and ethylene signal transduction and pronounced downregulation of hundreds of genes related to root hair development. Glucose-activated TOR signaling modulates the expression of root hair-specific (RHS) genes mainly through the core RHD6-RSL4 transcriptional cascade. The transcription of *RSL1*–*RSL5* was strongly dependent on functional TOR activity, whereas RHD6 transcript abundance was specifically induced by glucose–TOR signaling under carbon-starvation recovery conditions. Genetic overexpression of either *RHD6* or *RSL4* partially rescued root hair elongation defects caused by TOR suppression, confirming that the RHD6-RSL4 cascade functions as a critical downstream transcriptional module of glucose–TOR signaling. Collectively, this work establishes a transcriptional framework in which glucose–TOR signals modulate root hair elongation via transcriptional activation of the master bHLH regulators *RHD6* and *RSL4*.

## 1. Introduction

Root hairs, tubular outgrowths from root-specific epidermis, can greatly increase the root surface area and contribute to nutrient and water absorption, root anchoring ability, and root−microbe interactions [1]. In *Arabidopsis*, root hairs arise in a position-dependent pattern [2]. Specified root hair cells lie over clefts between two underlying cortical cells (the ‘‘H’’ cell position), and non-hair cells lie over one underlying cortical cell (the ‘‘N’’ cell position) in *Arabidopsis* [2,3]. In previous studies, several components involved in root epidermis cell fate determination were identified by using genetic and molecular approaches in *Arabidopsis* [2,4]. Among these components, *GL2* (*GLABRA 2*) plays a crucial role in maintaining N-cell fate and repressing root hair development. The loss-of-function mutant *gl2* displays root hairs at both the H and N positions [5]. A core regulatory complex including *TTG* (*TRANSPARENT TESTA GLABRA*), *WER* (*WEREWOLF*) and *GL3* (*GLABRA3*) or ENHANCER OF GLABRA3 (EGL3) can form the WER-GL3/EGL3-TTG transcriptional complex to regulate the expression of *GL2* and promote N-cell fate specification [6,7,8,9]. On the other hand, the cell-to-cell movement of CPC (CAPRICE) can repress *GL2* expression to maintain H cell fate in root epidermal cells through interactions with the TTG-WER-GL3 complex [9,10,11,12,13,14].

Once root hair cell fate is determined, root hair (trichoblast) initiation begins with localized swelling at the basal end of the cell in *Arabidopsis* [2,15]. A subset of the bHLH-type gene family downstream of *GL2* is important for root hair initiation and subsequent elongation. Among the genes in this family, *ROOT HAIR DEFECTIVE6* (*RHD6*) plays a major role in root hair morphogenesis, and the loss-of-function mutant *rhd6* displays a hairless phenotype [16]. In addition, *ROOT HAIR DEFECTIVE6-LIKE1* (*RSL1*), the closest homolog of *RHD6*, plays a partially redundant role with *RHD6* in promoting root hair initiation [17]. *RHD6* and *RSL1* can positively regulate four other genes in this family, *RSL2*–*RSL5*, to promote root hair initiation and elongation [18], and *RSL4* seems to be directly regulated by *RHD6* in *Arabidopsis* [19,20]. Previous studies have shown that *RHD6*/*RSLs* can regulate root hair growth by activating the expression of many root hair-specific (RHS) genes, including peroxidases, extensins and expansins [21,22,23]. Specifically, RSL4 was shown to induce the expression of many RHS genes by binding to root hair-specific *cis*-element (RHE) in their regulatory regions [21]. Furthermore, recent studies have shown that RHD6/RSLs are required for root hair growth induced by phytohormones, including auxin, ethylene and jasmonate [20,22,24]. Thus, a root hair morphogenesis regulatory network consisting of hormones, *RHD6*/*RSLs* and RHS genes is partially revealed.

Target of rapamycin (TOR), a conserved but atypical Ser/Thr protein kinase in all eukaryotes, belongs to the phosphatidylinositol 3-kinase-related lipid kinase family (PIKKs) [25]. TOR is a target of an immunosuppressant macrolide named rapamycin, which is produced by the soil bacterium *Streptomyces hygroscopicus* from Easter Island [26,27,28]. TOR can interact with several proteins to form two distinct complexes (target of rapamycin complex 1 (TORC1) and target of rapamycin complex 2 (TORC2)) to perform different functions in yeast and mammals [29]. Rapamycin, the first generation of TOR kinase inhibitors, can form a complex with the 12-kDa FK506-binding protein (FKBP12) to inhibit TORC1 activity [29]. In plants, TOR was first identified in *Arabidopsis* by Menand et al. in 2002, and only TORC1 has been found in plants [30,31]. Studies on TOR functions are often limited because of the embryo lethality of null *tor* mutants and the resistance of terrestrial plants to rapamycin [30,32]. Different strategies have been used to dissect the function of TOR in plants, including the generation of inducible *TOR* RNA interference (RNAi) lines, the overexpression of *FKBP12* in plants to rescue plant sensitivity to rapamycin, the use of second-generation TOR-specific chemical inhibitors (AZD8055, KU-63794, TORIN1 and TORIN2, etc.) and integrated multiomics analyses [17,33,34,35,36,37,38,39,40,41]. In the past few decades, accumulating evidence indicates that TOR can integrate nutrient or hormone signals such as sugars, auxin and abscisic acid (ABA) to balance growth and defense responses in plants [25]. Both Snf1-related kinase 1 (SnRK1) and Snf1-related kinase 2 (SnRK2) can phosphorylate regulatory-associated protein of TOR (RAPTOR) to respond to various stresses by inhibiting TOR activity [42,43]. Conversely, auxin can promote the activation of TOR to orchestrate plant growth and development through Rho-related GTPase from plants 2 (ROP2) [44,45]. Thus, the growth and development of plants can be precisely modulated by dynamic TOR activity through TOR-specific substrates. Some TOR substrates in *Arabidopsis* have been identified by different research groups, including ribosomal protein S6 kinase (S6K), type 2A phosphatase-associated protein 46 kDa (TAP46), E2F transcription factor A/B (E2FA/B), ABA receptors pyrabactin resistance 1-like (PYLs) and yet another kinase 1 (YAK1) [34,43,46,47,48]. TOR and its substrates form a complex regulatory network to regulate different biological processes. For instance, TOR regulates ribosomal protein S6 (RPS6) to control translation by modulating the phosphorylation of S6K in yeast, animals and plants [25,34,49]. Like in other eukaryotes, plant TAP46, an ortholog of TAP42 in yeast and α4 in animals, is a conserved regulator of protein phosphatase 2A (PP2A) activity, and TOR can modulate PP2A activity through TAP46 in *Arabidopsis* [46,50]. The TOR pathway is also involved in brassinosteroid signaling to regulate photoautotrophic development and hypocotyl growth in *Arabidopsis* [39,51]. In a recent study, Leene et al. screened many potential direct TOR substrates through proteomic, phosphoproteomic and interactomic analyses and proposed that the TOR-dependent complex regulatory network needs further study to address the fundamental roles of TOR in regulating cell growth and metabolism in plants [41].

Previous genetic evidence confirms that TOR positively regulates root hair elongation [35,40,52,53,54]; however, its underlying mechanism has not been fully characterized. Here, transcriptomic and genetic complementation assays were combined to elucidate TOR-dependent root hair transcriptional programs. Pharmacological TOR inhibition abolishes auxin- and ethylene-triggered root hair elongation, and hormone signaling mutants exhibit altered sensitivity to TOR suppressors, indicating that TOR operates alongside or downstream of these hormone signaling pathways. Further genetic and transcriptional evidence revealed that glucose–TOR signaling governs root hair development via transcriptional control of the RHD6-RSL4 cascade. The overexpression of either *RHD6* or *RSL4* partially rescued root hair elongation defects caused by rapamycin treatment. Collectively, the results of this study provide a transcriptional framework explaining how the glucose–TOR pathway promotes root hair growth through the activation of the master RHD6/RSL transcription factors in *Arabidopsis*.

## 2. Results

### 2.1. TOR Modulates Auxin and Ethylene Signaling During Root Hair Elongation

**Figure 1:**
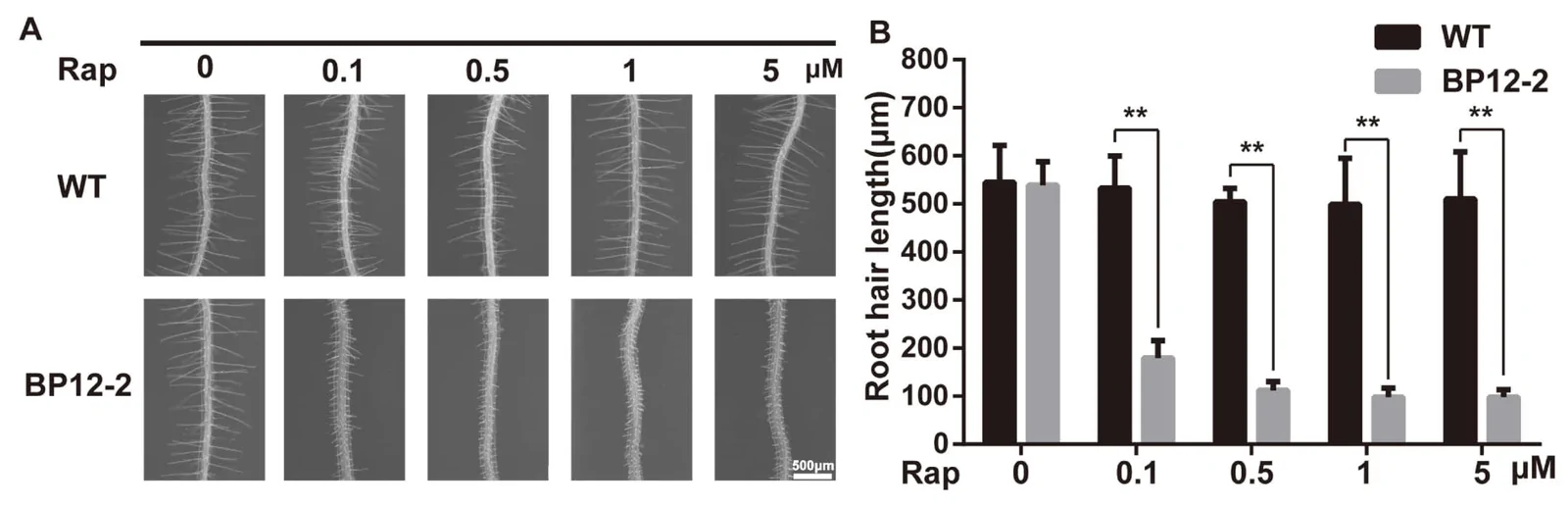
Rapamycin dose-dependently inhibited root hair elongation in BP12-2 transgenic *Arabidopsis* seedlings. (**A**) Stereomicrographs of root hair phenotypes of wild-type (Col, WT) and BP12-2 seedlings at five days after germination (Five-DAG) grown on 0.5× Murashige and Skoog (MS) medium supplemented with rapamycin (0 μM, 0.1 μM, 0.5 μM, 1 μM, or 5 μM). Bar = 500 μm. (**B**) Quantification of the root hair length of WT and BP12 seedlings under each rapamycin treatment. Two root hairs were measured from each of 10 independent seedlings per biological replicate, and individual seedlings represented the experimental unit. Multiple root-hair measurements from a single seedling were treated as subsamples. Three independent biological replicates were performed. The error bars represent the standard deviation. Statistical analysis was performed using two-way ANOVA followed by Tukey’s HSD multiple comparison test (** *p* < 0.01).

To further characterize the functions of TOR in root hair elongation in *Arabidopsis*, transgenic BP12-2 plants overexpressing yeast *FKBP12* were used for rapamycin-dependent TOR inhibition [35]. A dose gradient of rapamycin (100 nM to 5 μM) caused progressive shortening of root hairs in BP12-2 seedlings, whereas WT roots remained unaffected, confirming that BP12-2 is a reliable genetic system for investigating TOR function in root hair development (Figure 1).

Previous studies have shown that auxin and ethylene are two important hormones involved in promoting root hair elongation in *Arabidopsis* [4,55]. To investigate the relationship between TOR signaling and auxin/ethylene pathways in the regulation of root hair elongation, the root hair lengths of BP12-2 seedlings upon indole-3-acetic acid (IAA) or 1-aminocyclopropane-1-carboxylic acid (ACC) treatment were quantified. Exogenous IAA (auxin) and ACC (ethylene precursor) strongly stimulated root hair elongation in untreated BP12-2, but this growth promotion was abolished upon rapamycin treatment, demonstrating that TOR activity is required for hormone-induced root hair outgrowth (Figure S1).

To elucidate the genetic interaction between TOR and hormone signaling pathways, root hair lengths of hormone signaling-related plant materials were quantified following treatment with rapamycin or the ATP-competitive TOR inhibitor AZD8055. *TIR1* overexpression partially reversed the rapamycin-induced root hair defects in the BP12-2 background (Figure S2A,B) [40]. In contrast, the *tir1 afb1 afb2 afb3* quadruple auxin receptor mutant exhibited extreme hypersensitivity to AZD8055 (Figure S2C,D). A forward genetic screen revealed hormone mutants with altered TOR inhibitor sensitivity. The gain-of-function *axr2* (*IAA7*) auxin signaling mutant presented severely shortened root hairs upon AZD8055 exposure, whereas the *ctr1* constitutive ethylene response mutant was largely insensitive to TOR suppression. Ethylene signaling null mutants *ein2-1* and *ein3eil1* mirrored the hypersensitive phenotype of the auxin receptor quadruple mutants (Figure S2C–F). Collectively, these pharmacological and genetic phenotypic analyses support that TOR acts genetically downstream of or in parallel to canonical auxin and ethylene transcriptional cascades to modulate root hair elongation.

### 2.2. TOR Globally Reprograms the Root Transcriptome to Control Root Hair Development Programs

To elucidate the role of TOR in root hair elongation, RNA sequencing (RNA-seq) was performed on rapamycin- and dimethyl sulfoxide (DMSO)-treated BP12-2 seedling roots to identify TOR-dependent transcriptional networks. A total of 4602 differentially expressed genes (DEGs) were identified between the two treatment groups, among which 1985 genes were upregulated and 2617 genes were downregulated (Table S1, Figure S3). Enrichment analysis revealed widespread transcriptional repression of auxin biosynthesis, transport, and signal transduction genes, along with downregulation of ethylene metabolic and signaling components (Figure S4), which is consistent with phenotypic data linking TOR to hormone-stimulated root hair growth.

**Figure 2:**
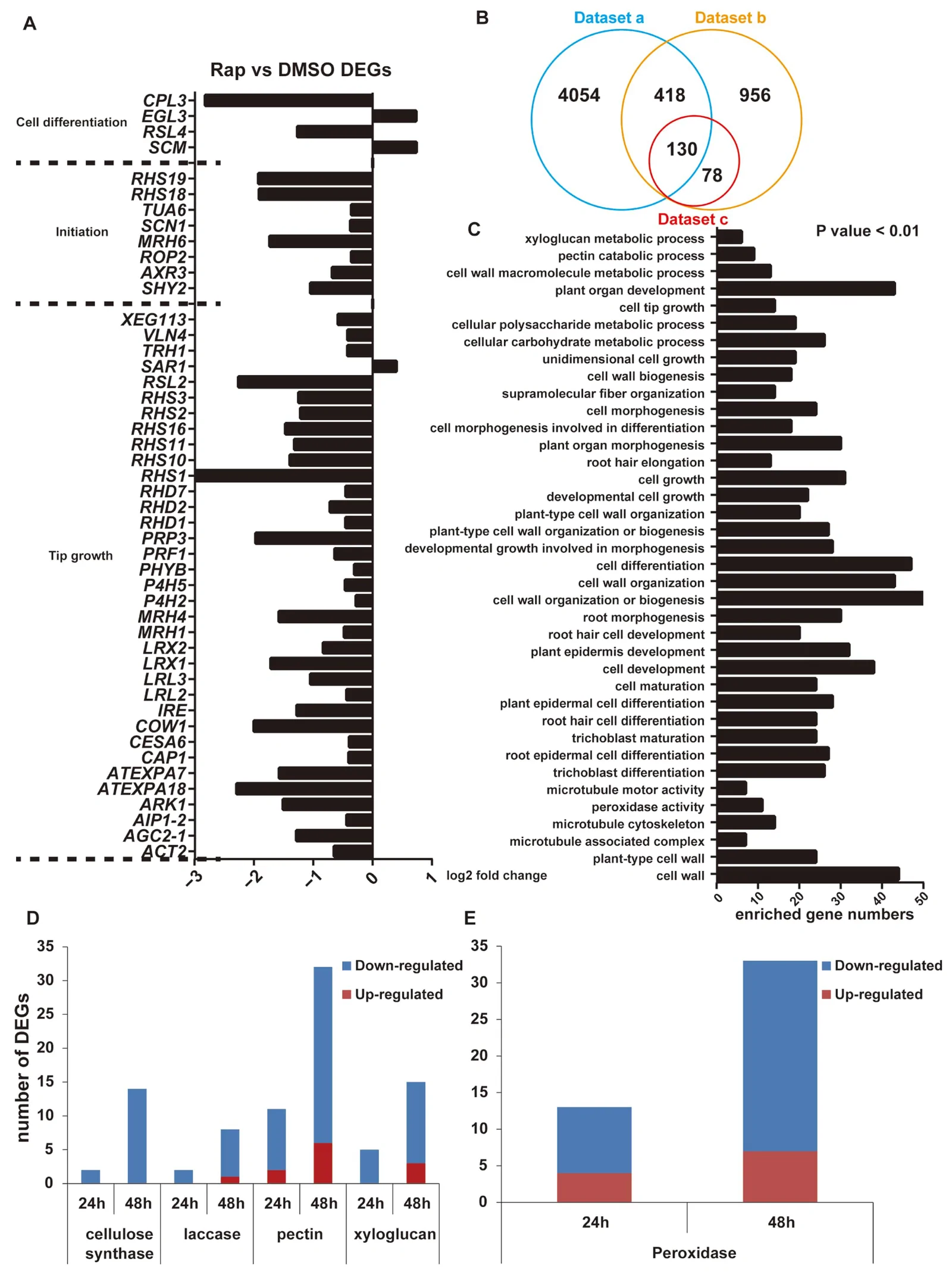
TOR inhibition triggers broad transcriptional reprogramming of root hair development genes in *Arabidopsis* seedling roots. RNA-seq was performed on BP12-2 roots treated with 5 μM rapamycin or the DMSO control for 24 h and 48 h. (**A**) Bar plot illustrating log_2_-fold-change values of representative TOR-regulated genes grouped by three root hair developmental stages (cell differentiation, initiation, and tip growth) after 48 h rapamycin treatment compared with the DMSO control. Bar height corresponds to the log_2_ fold change in individual genes. (**B**) Venn diagram illustrating the three gene datasets. Dataset a corresponds to 4602 rapamycin-responsive DEGs identified in this study. Dataset b corresponds to 1582 root hair-related DEGs identified by Bruex et al. (Table S3A) [57]. Dataset c corresponds to the 208 core genes related to root hair development identified by Bruex et al. (Table S3B) [57]. (**C**) Enriched Gene Ontology (GO) biological process terms derived from the 548 shared DEGs presented in Panel B. Terms associated with root hair development are displayed, with the significance threshold set at an adjusted *p*-value < 0.01. Full adjusted *p*-values for each GO term are provided in Table S4. The height of each bar indicates the number of differentially expressed genes annotated to each GO biological-process term. (**D**) Number of differentially expressed genes encoding cell wall synthases and modifiers upon 24 h and 48 h of rapamycin treatment. The blue bars indicate downregulated genes, and the red bars indicate upregulated genes. (**E**) Counts of differentially expressed peroxidase genes detected after 24 h and 48 h of rapamycin treatment. The blue bars indicate downregulated genes, and the red bars indicate upregulated genes.

Previous studies using forward- and reverse-genetics approaches have identified numerous genes influencing root hair growth, which are cataloged in the iRootHair database [56]. The DEG dataset was cross-referenced with 138 known root hair regulatory genes curated from the iRootHair database (Table S2A) [56]. Fifty-seven overlapping genes were identified and covered all three major developmental stages, including epidermal cell fate specification, root hair initiation and tip growth (Figure 2A and Table S2B). The majority of these shared genes were transcriptionally repressed by rapamycin and enriched for tip growth functions (Figure 2A and Table S2B). The transcriptome dataset was further integrated with a published microarray dataset comparing hairy and hairless *Arabidopsis* mutants, yielding 548 coregulated DEGs (Figure 2B, Table S3) [57]. GO enrichment analysis of these shared genes highlighted core biological processes such as cell wall organization, cell expansion, root hair differentiation and polar tip growth (Figure 2C, Table S4). Bruex et al. previously defined 208 core root epidermal marker genes [57]. Nearly two-thirds of these marker genes overlapped with the TOR-regulated DEGs identified in this study, most of which are highly expressed in trichoblasts (Table S3B), positioning TOR as a master transcriptional regulator of root epidermal identity.

Cell wall remodeling and reactive oxygen species (ROS) production are essential for root hair tip growth [24,57,58]. Rapamycin significantly repressed the expression of genes encoding cellulose synthases, pectin-modifying enzymes, laccases, and xyloglucan endotransglucosylases, indicating that TOR controls the transcription of key cellulose and pectin biosynthetic machinery (Figure 2D,E, Table S5). The expression of peroxidase genes, which are critical for ROS homeostasis during polar growth, was also altered, with 28 peroxidase genes downregulated and 8 upregulated following TOR inhibition (Figure 2E, Table S5). Additionally, the transcription of *root hair defective 2* (*RHD2*), the NADPH oxidase required for ROS generation in growing root hairs, was strongly suppressed by rapamycin (Table S1) [59]. These transcriptomic signatures confirm that TOR orchestrates the full transcriptional program supporting root hair elongation.

To further explore the potential upstream transcriptional regulators mediating TOR-dependent root hair development, all the transcription factors within the 4602 rapamycin-responsive DEGs were systematically annotated. In total, 304 transcription factors (TFs) exhibited significantly altered transcript abundance upon TOR inhibition (Table S1, Figure S5). To further dissect cell type-specific transcriptional regulators downstream of TOR, published *Arabidopsis* root single-cell RNA-seq datasets were integrated to profile the expression patterns of all 304 TOR-responsive TFs identified from the rapamycin-treated transcriptome [60]. Unsupervised clustering partitioned root cells into discrete subpopulations, among which Clusters 8 and 17 were annotated as trichoblasts (root hair precursor cells). Consistent with cell-type specialization, a subset of these TFs exhibited prominent transcriptional enrichment within trichoblast clusters (Figure S6A). Cross-dataset coexpression overlap analysis was subsequently performed to link these trichoblast-enriched TFs with the full set of TOR-regulated genes identified by RNA-seq. The coexpression networks revealed extensive overlap between the targets of these trichoblast-enriched TFs and TOR-responsive DEGs, supporting a role for these TFs in mediating TOR-dependent root hair development (Figure S6B). Follow-up functional experiments on these poorly studied root hair TFs will help to fully elucidate the multilayered transcriptional network modulated by TOR in *Arabidopsis* roots.

### 2.3. The RHD6-RSL4 Transcriptional Cascade Acts Downstream of Glucose–TOR Signaling

Notably, RSL4, a key transcription factor known to govern root hair growth, was among the core genes closely coexpressed with these trichoblast-specific TFs (Figure S6). These observations point to a potential regulatory connection between TOR signaling and the RSL4-centered root hair transcriptional program. Further genetic and molecular experiments were therefore performed to investigate the functional relationship between TOR and the RHD6-RSL4 regulatory module during root hair elongation.

**Figure 3:**
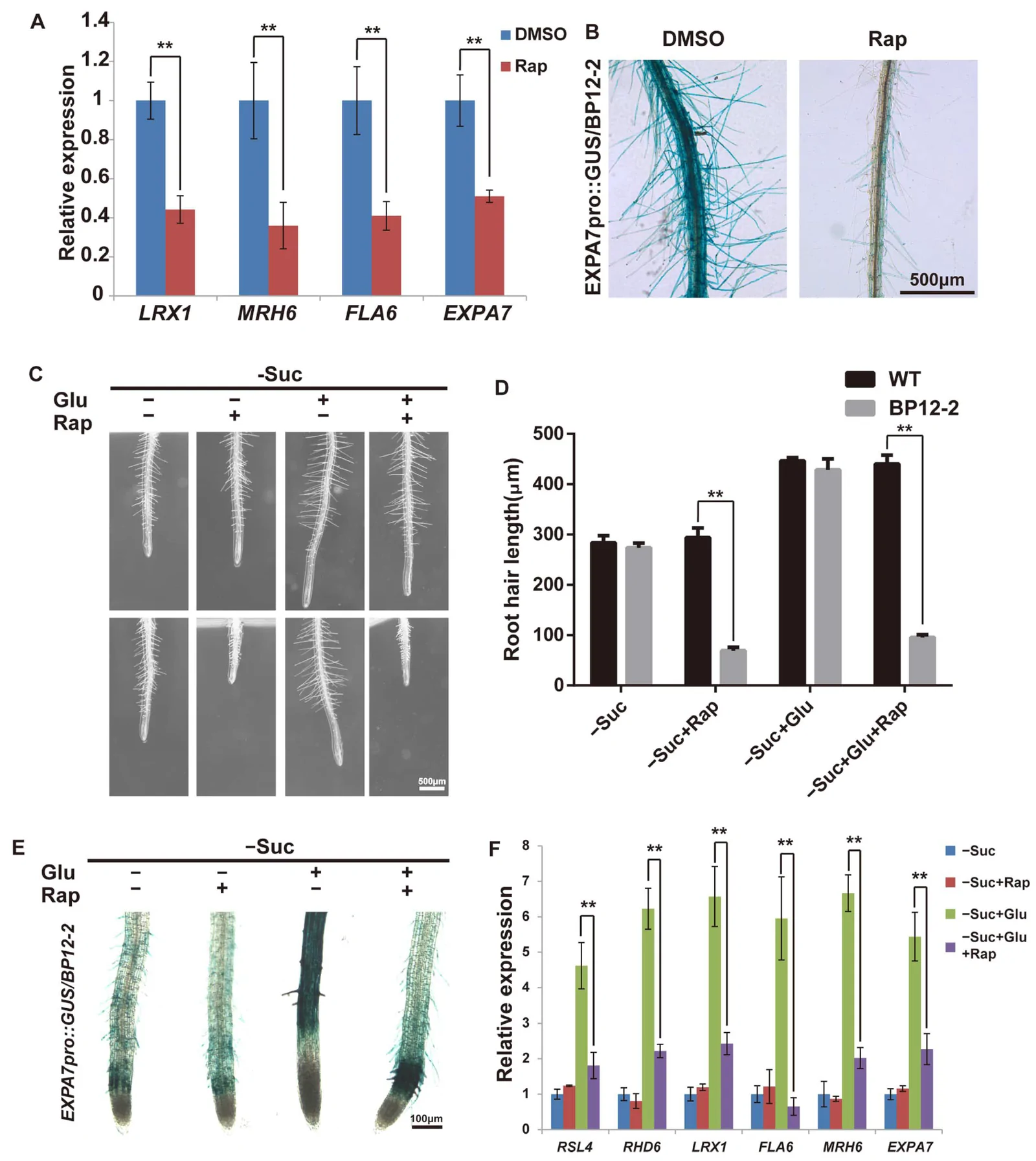
Glucose-activated TOR signaling drives the transcription of RHS genes via the RHD6-RSL4 module. (**A**) RT–qPCR quantification of representative RHS gene transcript levels in BP12-2 roots treated with 5 μM rapamycin or DMSO for 24 h. Five-DAG BP12-2 seedlings grown on 0.5× MS medium were transferred to medium supplemented with 5 μM rapamycin or DMSO for 24 h under standard growth conditions, after which the root tissues were harvested for RNA isolation and RT–qPCR analysis. (**B**) GUS reporter activity of EXPA7pro::GUS/BP12-2 upon rapamycin treatment. Five-DAG EXPA7pro::GUS/BP12-2 seedlings grown on 0.5× MS medium were transferred to medium supplemented with 5 μM rapamycin or DMSO for 24 h under standard growth conditions, after which they were subjected to GUS staining. Scale bar = 500 μm. Three independent biological replicates were performed, and more than 10 individual seedlings were inspected for each treatment within each replicate to confirm consistent staining patterns. (**C**) Stereomicrographs showing the root-hair phenotypes of WT and BP12-2 seedlings cultured in the presence or absence of 30 mM glucose and 5 μM rapamycin for 48 h. Five-DAG WT and BP12-2 seedlings grown on sugar-free 0.5× MS medium were transferred to sugar-free medium or medium supplemented with 30 mM glucose, together with 5 μM rapamycin or DMSO, for 48 h under standard growth conditions prior to stereomicroscopic observation of the roots. Scale bar = 500 μm. (**D**) Quantitative measurement of root-hair length corresponding to Panel C. Two root hairs were measured from each of 10 independent seedlings per biological replicate, and individual seedlings represented the experimental unit. Multiple root-hair measurements from a single seedling were treated as subsamples. Three independent biological replicates were performed. (**E**) GUS staining of EXPA7pro::GUS/BP12-2 under combined glucose and rapamycin treatment for 24 h. Five-DAG EXPA7pro::GUS/BP12-2 seedlings grown on sugar-free 0.5× MS medium were transferred to sugar-free medium or medium supplemented with 30 mM glucose, together with 5 μM rapamycin or DMSO, for 24 h under standard growth conditions, after which the samples were harvested for GUS staining. Scale bar = 100 μm. Three independent biological replicates were performed, and more than 10 individual seedlings were inspected for each treatment within each replicate to confirm consistent staining patterns. (**F**) RT-qPCR analysis of the transcript abundance of the *RHD6*, *RSL4*, and core RHS genes under the conditions of sugar starvation, glucose supplementation, and glucose treatment with or without 5 μM rapamycin. Five-DAG BP12-2 seedlings grown on sugar-free 0.5× MS medium were transferred to sugar-free medium or medium supplemented with 30 mM glucose, together with 5 μM rapamycin or DMSO, for 24 h under standard growth conditions. Root tissues were harvested for RNA extraction and RT–qPCR analysis. The error bars represent the standard deviation. Statistical analysis was performed using two-way ANOVA followed by Tukey’s HSD multiple comparison test. Only pre-planned pairwise comparisons are marked in the figure (** *p* < 0.01).

RHS genes constitute a suite of transcripts that are exclusively enriched in trichoblasts, whose transcription is predominantly controlled by the RHD6-RSL4 regulatory module and whose core functions support root hair morphogenesis [19,21,61]. In this study, transcriptome data revealed that the expression of multiple canonical RHS genes was significantly downregulated in the roots of rapamycin-treated BP12-2 plants (Table S6). RT–qPCR validated this transcriptional repression of representative RHS family members (Figure 3A and Figure S7). *EXPA7* is a typical RHS gene and is predominantly expressed in root hairs in *Arabidopsis* [62]. *EXPA7* promoter-driven *β-glucuronidase* (*GUS*) reporter gene lines in the BP12-2 background were generated, and histochemical staining revealed robust loss of *EXPA7* promoter activity in root hairs after rapamycin exposure (Figure 3B). Previous studies have shown that glucose is a primary activating signal for plant TOR in *Arabidopsis* [34,52]. In sugar-free growth medium, exogenous glucose strongly induced root hair elongation in WT seedlings, but this stimulatory effect was fully blocked by rapamycin cotreatment (Figure 3C,D). Similarly, glucose treatment robustly activated the transcription of RHS genes, and this induction was eliminated upon TOR inhibition (Figure 3E,F), establishing glucose–TOR signaling as a positive transcriptional regulator of root hair-specific gene programs.

**Figure 4:**
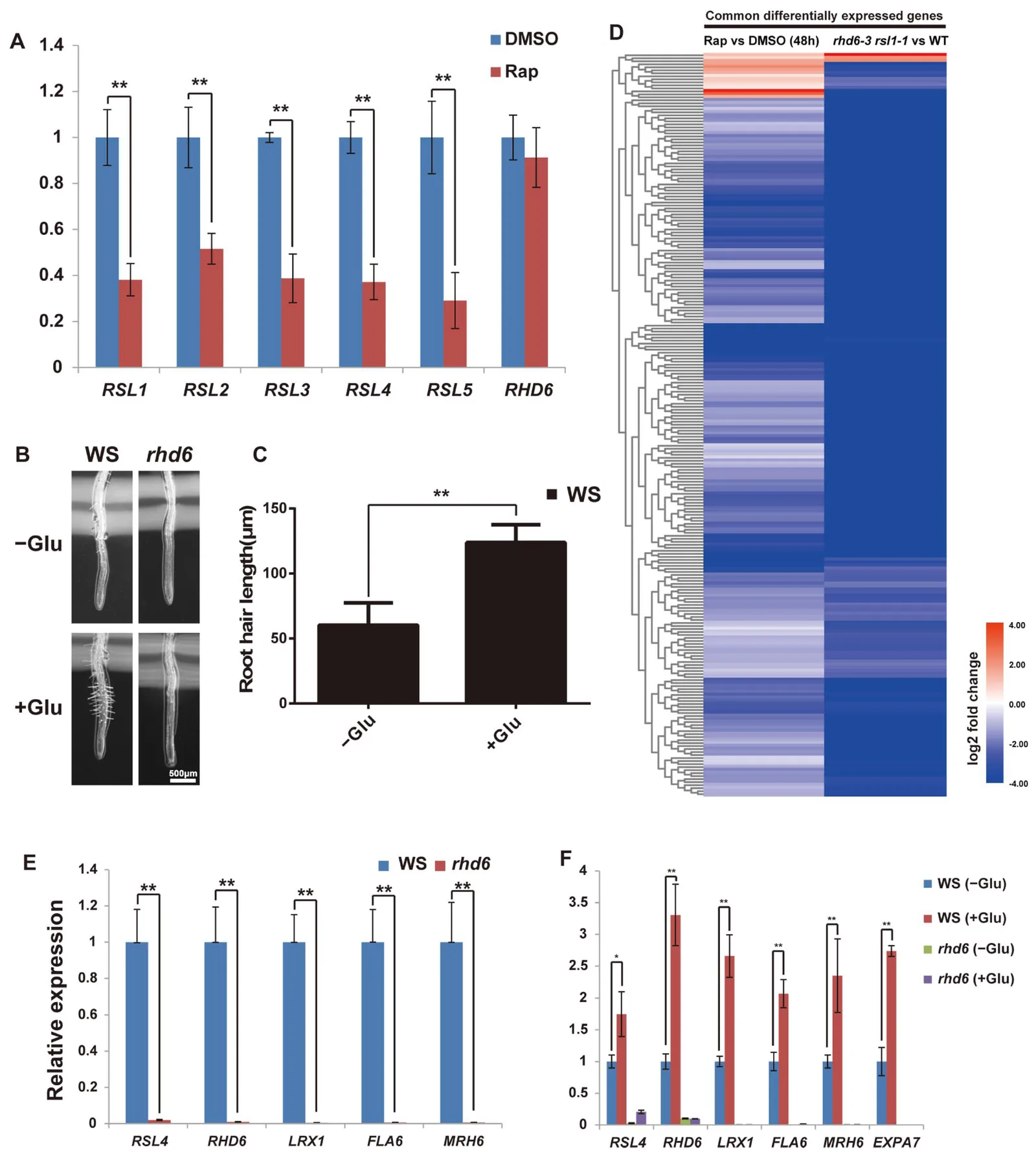
Glucose-induced root hair elongation is dependent on functional *RHD6*. (**A**) RT-qPCR detection of the transcript abundance of *RSL1*–*RSL5* and *RHD6* in BP12-2 roots treated with 5 μM rapamycin or DMSO for 24 h. Five-DAG BP12-2 seedlings grown on 0.5× MS medium were transferred to medium supplemented with 5 μM rapamycin or DMSO for 24 h under standard growth conditions, after which the root tissues were harvested for RNA extraction and RT-qPCR analysis. (**B**) Root-hair phenotypes of WS (Wassilewskija ecotype) and *rhd6* (WS background) mutant seedlings cultured in the presence or absence of 30 mM glucose for 24 h. Five-DAG seedlings grown on sugar-free 0.5× MS medium were transferred to sugar-free medium or medium supplemented with 30 mM glucose for 24 h under standard growth conditions prior to stereomicroscopic observation of the roots. Scale bar = 500 μm. (**C**) Quantification of root-hair length for WS seedlings corresponding to Panel B. Two root hairs were measured from each of 10 independent seedlings per biological replicate, and individual seedlings represented the experimental unit. Multiple root-hair measurements from a single seedling were treated as subsamples. Three independent biological replicates were performed. (**D**) Heatmap showing log_2_ fold-change values of co-differentially expressed genes. This gene set comprises genes significantly differentially expressed both in BP12-2 seedlings after 48 h rapamycin treatment relative to DMSO control, and in *rhd6-3 rsl1-1* double-mutant seedlings relative to WT wild-type. Data were transformed to log_2_ fold-change. The complete colour scale ranges from −4.00 (blue, down-regulated genes) to +4.00 (red, up-regulated genes). The full gene list is provided in Table S7. (**E**) RT-qPCR analysis of the transcript levels of *RHD6*, *RSL4*, and representative RHS genes in WS and *rhd6* seedlings under normal growth conditions. The roots of Five-DAG seedlings that had grown on 0.5× MS medium were collected for RNA extraction and RT–qPCR. (**F**) RT-qPCR analysis of the transcript levels of *RHD6*, *RSL4*, and representative RHS genes in WS and *rhd6* seedlings under sugar-free conditions or after 24 h of induction with 30 mM glucose. Five-DAG seedlings grown on sugar-free 0.5× MS medium were transferred to sugar-free medium or medium supplemented with 30 mM glucose for 24 h under standard growth conditions, after which the root tissues were harvested for RNA extraction and RT-qPCR analysis. The error bars represent the standard deviation. Statistical analysis was performed using two-way ANOVA followed by Tukey’s HSD multiple comparison test. Only pre-planned pairwise comparisons are marked in the figure (* *p* < 0.05, ** *p* < 0.01).

Given the central role of RHD6/RSL bHLH factors in activating RHS transcription [16,18,19,21], this study examined whether TOR affects the expression of these RHS genes through the RHD6/RSL module. To test this hypothesis, RT–qPCR analyses were performed to measure the transcript levels of RHD6/RSLs in BP12-2 seedlings with or without rapamycin treatment. The results revealed that the *RSL1–RSL5* transcript level significantly decreased after rapamycin treatment, whereas the RHD6 transcript level remained unaltered under standard growth conditions (Figure 4A). Transcriptomic data independently confirmed the significant downregulation of *RSL2*, *RSL4*, and *RSL5* upon TOR suppression (Table S1).

Previous studies have demonstrated that the expression of *RSL2*–*RSL5* is transcriptionally regulated by the upstream factors RHD6 and RSL1 and that the *rhd6* loss-of-function mutant presents a complete hairless phenotype [16,19], suggesting that RHD6 acts as a core regulator of the root hair elongation process. To investigate the role of RHD6 in glucose–TOR signaling-mediated root hair elongation, the root hair phenotypes of wild-type (WS) and *rhd6-1* mutant seedlings were compared under glucose-supplemented and glucose-free medium conditions. The results revealed that glucose-dependent root hair elongation is fully RHD6 dependent. The wild-type (*WS*) control seedlings developed elongated root hairs when supplemented with glucose, whereas the *rhd6* seedlings remained hairless regardless of the presence of sugar (Figure 4B,C). Furthermore, differentially expressed genes were compared between rapamycin-treated BP12-2 seedlings and the *rhd6-3 rsl1-1* double mutant. The data revealed that 247 genes were coregulated by TOR inhibition and loss of RHD6-RSL1 function (Figure 4D, Table S7). GO enrichment of the 232 shared downregulated RHS genes revealed exclusive enrichment for root hair growth pathways (Table S8). The expression of *RHD6* and these 232 shared downregulated RHS genes was further examined in both wild-type (WS) and *rhd6* mutant plants. In the *rhd6* mutant, RHS genes were not detectable under normal conditions. While glucose treatment robustly induced RHS gene expression in WS plants, this induction was almost completely abolished in the *rhd6* mutant background (Figure 4E,F). These data indicate that *RHD6* is an important node of glucose–TOR signaling in the regulation of root hair growth.

**Figure 5:**
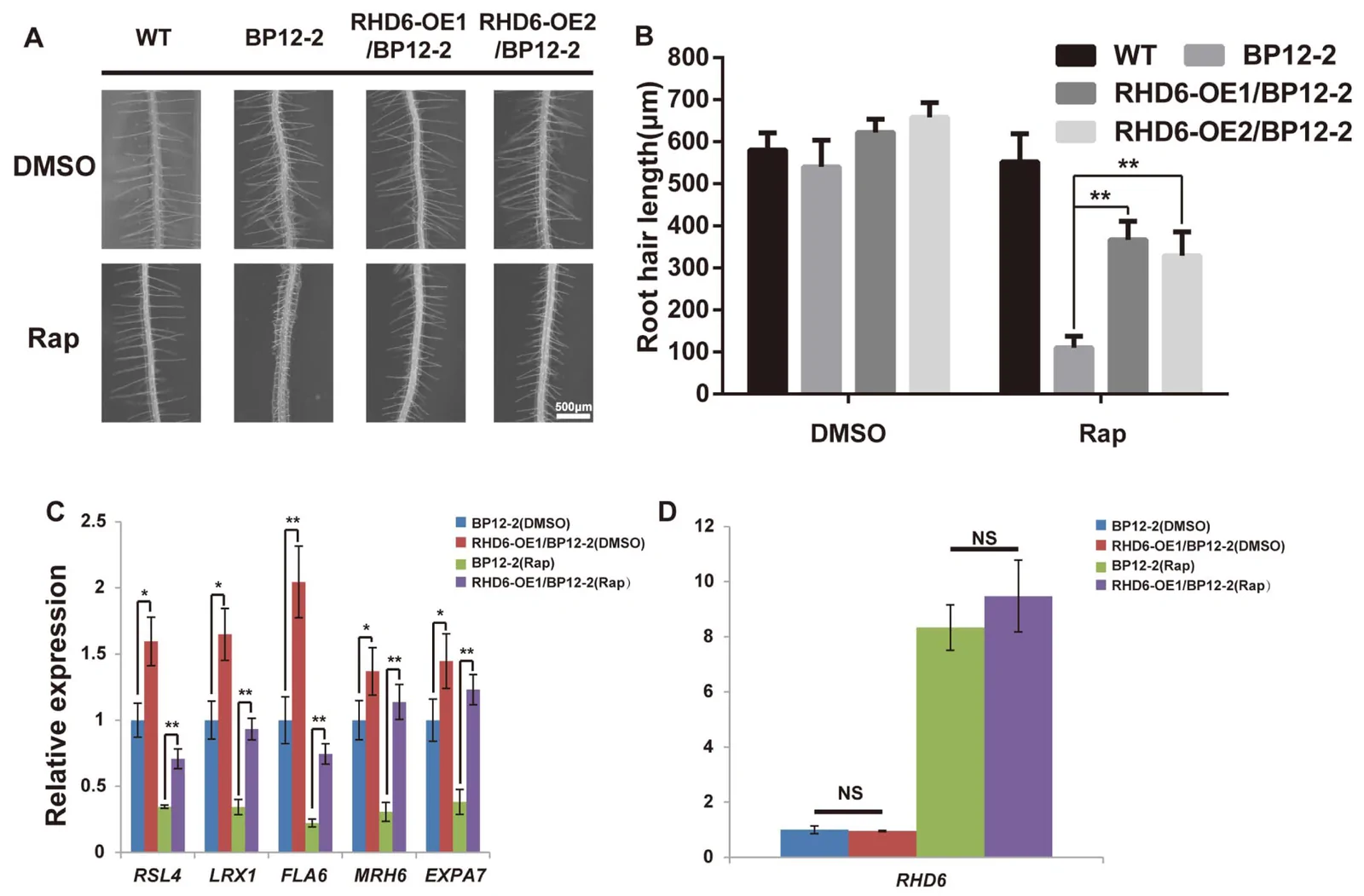
Overexpression of *RHD6* partially reversed the rapamycin-induced defects in root hair elongation in the BP12-2 background. (**A**) Stereomicrographs showing the root-hair phenotypes of WT, BP12-2, RHD6-OE1/BP12-2 and RHD6-OE2/BP12-2 plants under DMSO and rapamycin treatment. Scale bar = 500 μm. Seeds of the WT, BP12-2 and RHD6OE/BP12-2 lines were subsequently grown on 0.5× MS medium supplemented with 5 μM rapamycin or DMSO until Five-DAG, after which the root-hair phenotypes were observed by stereomicroscopy. (**B**) Quantification of root hair length corresponding to Panel A. Two root hairs were measured from each of 10 independent seedlings per biological replicate, and individual seedlings represented the experimental unit. Multiple root-hair measurements from a single seedling were treated as subsamples. Three independent biological replicates were performed. (**C**) RT–qPCR detection of *RSL4* and representative RHS gene expression in BP12-2 and RHD6-OE1/BP12-2 seedlings with/without 5 μM rapamycin for 24 h. Five-DAG BP12-2 and RHD6-OE1/BP12-2 seedlings grown on 0.5× MS medium were transferred to medium supplemented with 5 μM rapamycin or DMSO for 24 h under standard growth conditions, after which the root tissues were harvested for RNA extraction and RT-qPCR analysis. (**D**) Relative *RHD6* transcript levels in BP12-2 and RHD6-OE1/BP12 roots. Five-DAG BP12-2 and *RHD6-OE1/BP12-2* seedlings grown on 0.5× MS medium were transferred to medium supplemented with 5 μM rapamycin or DMSO for 24 h under standard growth conditions, after which the root tissues were harvested for RNA extraction and RT-qPCR analysis. The error bars represent the standard deviation. Statistical analysis was performed using two-way ANOVA followed by Tukey’s HSD multiple comparison test. Only pre-planned pairwise comparisons are marked in the figure (* *p* < 0.05, ** *p* < 0.01, NS, not significant).

To genetically validate RHD6 as a downstream transcriptional effector of TOR, *RHD6* overexpression lines in the BP12-2 background (RHD6-OE/BP12-2) were generated. Under rapamycin treatment, RHD6-OE/BP12-2 seedlings exhibited substantially longer root hairs relative to empty BP12-2 controls (Figure 5A,B). RT-qPCR analysis showed that the rapamycin-mediated repression of *RSL4* and downstream RHS genes was markedly attenuated in *RHD6* overexpression lines (Figure 5C). Although *RHD6* transcript abundance was insensitive to rapamycin under a constant supply of sugar (Figure 5D), the addition of glucose after carbon starvation robustly induced *RHD6* expression in a TOR-dependent manner (Figure 4F).

**Figure 6:**
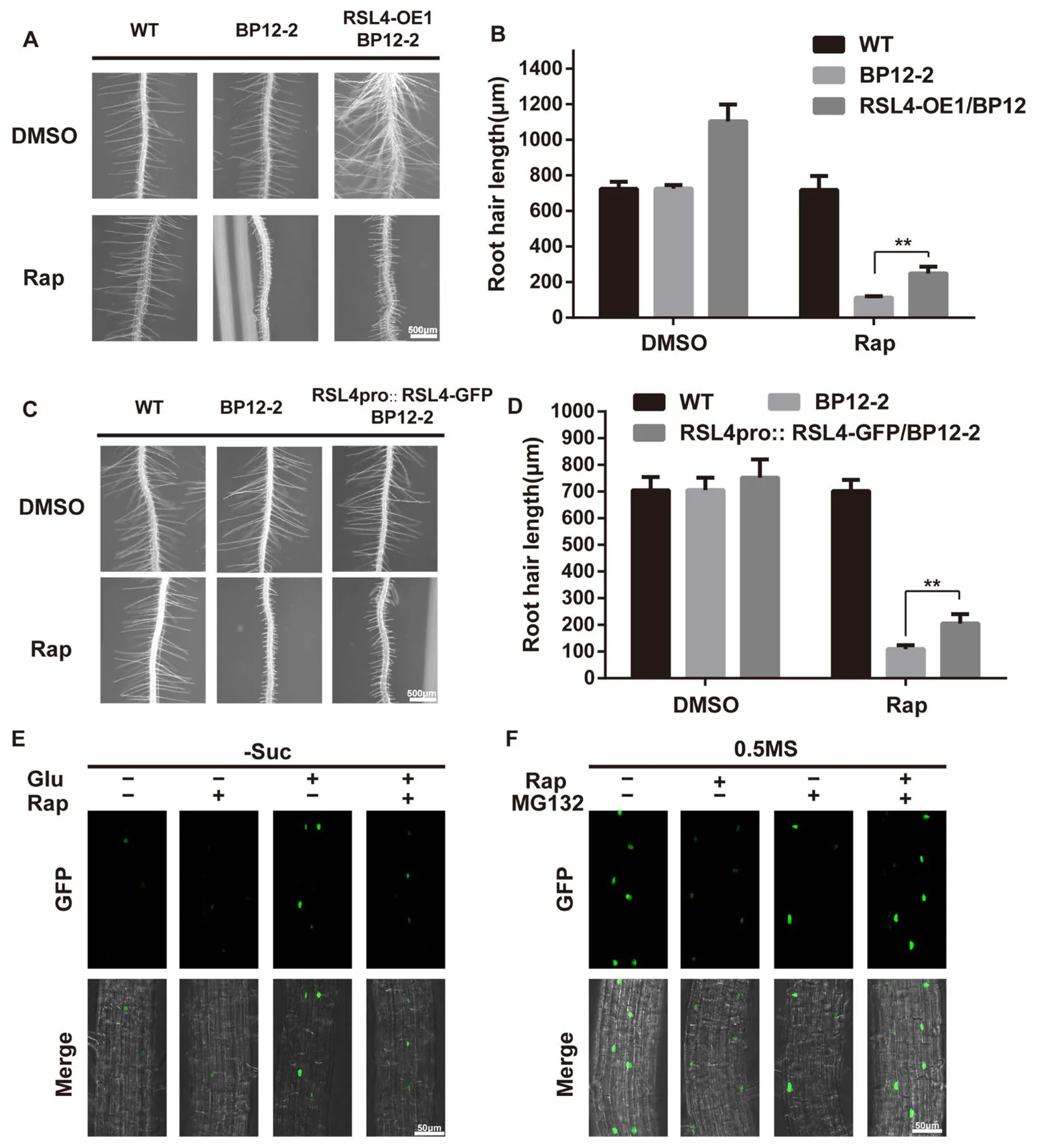
Glucose–TOR signaling promotes the accumulation of the RSL4 protein in trichoblasts. (**A**) Root-hair phenotypes of WT, BP12-2 and *RSL4-OE1/BP12-2* seedlings at Five-DAG following treatment with 5 μM rapamycin or DMSO. Seeds of the WT, BP12-2 and RSL4-OE1/BP12-2 lines were grown on 0.5× MS medium supplemented with 5 μM rapamycin or DMSO until Five-DAG, and root-hair phenotypes were observed by stereomicroscopy. Scale bar = 500 μm. (**B**) Quantification of root-hair length corresponding to Panel A. Two root hairs were measured from each of 10 independent seedlings per biological replicate, and individual seedlings represented the experimental unit. Multiple root-hair measurements from a single seedling were treated as subsamples. Three independent biological replicates were performed. (**C**) Root-hair phenotypes of WT, BP12-2 and RSL4pro::RSL4-GFP/BP12-2 reporter seedlings under rapamycin or DMSO treatment. Seeds of the WT, BP12-2 and RSL4pro::RSL4-GFP/BP12-2 lines were grown on 0.5× MS medium supplemented with 5 μM rapamycin or DMSO until Five-DAG, and root-hair phenotypes were observed by stereomicroscopy. Scale bar = 500 μm. (**D**) Quantification of root hair length corresponding to Panel C. Two root hairs were measured from each of 10 independent seedlings per biological replicate, and individual seedlings represented the experimental unit. Multiple root-hair measurements from a single seedling were treated as subsamples. Three independent biological replicates were performed. (**E**) Confocal laser-scanning micrographs showing RSL4-GFP fluorescence in root trichoblasts. Five-DAG RSL4pro::RSL4-GFP/BP12-2 seedlings grown on sugar-free 0.5× MS medium were transferred to sugar-free medium or medium supplemented with 30 mM glucose, with or without 5 μM rapamycin, for 24 h under standard growth conditions before confocal observation of RSL4-GFP signals. Scale bar = 50 μm. Representative micrographs are shown from three independent biological replicates. (**F**) Confocal laser-scanning micrographs of RSL4-GFP fluorescence in root trichoblasts under rapamycin and MG132 treatment. Five-DAG RSL4pro::RSL4-GFP/BP12-2 seedlings grown on 0.5× MS medium were transferred to medium supplemented with 5 μM rapamycin in the presence or absence of 10 μM MG132 for 24 h under standard growth conditions prior to confocal imaging of RSL4-GFP. Scale bar = 50 μm. Representative micrographs are shown from three independent biological replicates. The error bars represent the standard deviation. Statistical analysis was performed using two-way ANOVA followed by Tukey’s HSD multiple comparison test. Only pre-planned pairwise comparisons are marked in the figure (** *p* < 0.01).

In a previous study, Hwang et al. reported that many root hair-related genes, including RHS genes, were directly regulated by RSL4, a homolog of RHD6 [21]. *RSL4*, which was directly activated by RHD6, can sufficiently promote root hair growth independent of *RHD6* [19]. RSL4 was also found to be involved in downstream auxin and ethylene signaling to regulate root hair growth in *Arabidopsis* [22,24]. RSL4-overexpressing transgenic lines in the BP12-2 background (RSL4-OE/BP12-2 lines) were also generated. Under normal growth conditions, RSL4-OE/BP12-2 seedlings produced dense, elongated root hairs. Upon rapamycin treatment, *RSL4* overexpression partially restored root hair elongation defects relative to that observed in unmodified BP12-2 seedlings (Figure 6A,B).

In a previous study, Yi et al. reported that the accumulation of RSL4 in trichoblasts (future hair cells) could affect the final root hair length in *Arabidopsis* [19]. Compared with BP12-2 seedlings, RSL4-OE1/BP12-2 seedlings could have longer root hairs under rapamycin treatment, suggesting that rapamycin may affect RSL4 accumulation in trichoblasts to regulate root hair length in BP12-2. To determine whether TOR affects RSL4 protein accumulation, RSL4pro::RSL4-GFP/BP12-2 lines were generated. The results revealed that the root hairs of the RSL4pro::RSL4-GFP/BP12-2 line were also longer than those of BP12-2 under rapamycin treatment (Figure 6C,D).

Upon TOR inhibition, the fluorescence intensity of RSL4-GFP was markedly reduced in trichoblasts (Figure S8). Consistent with this, under sugar-free conditions, only weak RSL4-GFP signals were detectable in trichoblasts. Glucose treatment strongly promoted RSL4 accumulation, whereas this glucose-driven induction was largely abolished by rapamycin. Collectively, these observations indicate that glucose–TOR signaling plays a critical role in supporting RSL4 accumulation within trichoblasts, predominantly by stimulating RSL4 transcription (Figure 6E,F; Figure S9). An exploratory assay with the 26S proteasome inhibitor MG132 was further conducted to assess the proteasome-mediated turnover of the RSL4 protein. As shown in Figure 6F, MG132 treatment increased the accumulation of the RSL4-GFP line in trichoblasts, indicating that the RSL4 protein is subjected to proteasome-mediated degradation in vivo. Notably, this experiment cannot determine whether TOR directly regulates RSL4 protein turnover, as the reduced RSL4-GFP signal observed under rapamycin treatment can be largely accounted for by the robust transcriptional downregulation of *RSL4* at the transcriptional level.

Collectively, these genetic and transcriptional data demonstrate that TOR signaling modulates the RHD6-RSL4 module at the transcriptional level and that this transcriptional cascade is critical for TOR-mediated root hair elongation. Loss of TOR activity represses the transcription of RSL family genes and downstream RHS targets, whereas increasing RHD6 or RSL4 transcript levels via genetic overexpression can partially bypass TOR inhibition and restore root hair elongation.

## 3. Discussion

TOR acts as a conserved central integrator of cellular energy, nutrient, hormone, and stress cues, reshaping genome-wide transcription, translation, and metabolism to coordinate plant growth [25]. This study demonstrated that the RHD6-RSL4 transcriptional cascade is a critical downstream module that mediates TOR regulation of root hair elongation in *Arabidopsis*. Chemical suppression of TOR abolishes transcriptional activation of the RHD6-RSL4 cascade, which in turn eliminates the expression of hundreds of RHS genes and arrests root hair outgrowth. A combination of phenotypic, transcriptomic, and genetic data revealed that glucose-activated TOR drives root hair morphogenesis by maintaining robust expression of the master bHLH transcription factors *RHD6* and *RSL4*. In parallel, TOR inhibition broadly perturbs the transcript levels of auxin and ethylene biosynthesis and signaling components, which may act in concert with the RHD6-RSL4 cascade to modulate root hair growth.

In recent studies, the close relationship between glucose–TOR signaling and the auxin/ethylene pathway has been partly revealed by several research groups [40,44,45,63,64]. For instance, a recent study demonstrated that TOR modulates root system development by regulating the protein stability of PIN2, a key auxin efflux carrier required for polar auxin transport to root hair cells [65,66], suggesting a potential mechanism through which TOR influences root hair development via polar auxin transport. With respect to ethylene, TOR has been shown to regulate hypocotyl growth by modulating the function of 1-aminocy-clopropane-1-carboxylate (ACC) synthase (ACS2 and ACS6), the rate-limiting enzyme for ethylene biosynthesis [67]. In addition to the canonical ethylene-CTR1-EIN2 signaling cascade, TOR can also regulate the subcellular localization of EIN2 via phosphorylation and thereby control plant cell elongation and proliferation in a canonical ethylene signaling-independent manner [68].

Auxin and ethylene are well established positive regulators of root hair elongation [4,22,24]. Root hair outgrowth triggered by nutrient deficiency is largely mediated by auxin and ethylene signaling, representing a core adaptive strategy for plants to cope with nutrient limitation [69,70,71]. However, the molecular linkage connecting energy availability to these hormonal cascades in root hair development remained poorly defined prior to this work. Consistent with the documented multilayered crosstalk between TOR and hormone pathways, genetic data from this study revealed that overexpression of the auxin receptor *TIR1* partially rescued the root hair defects caused by TOR inhibition and that hormone signaling mutants displayed altered sensitivity to TOR inhibitors (Figure S2). Transcriptomic data further revealed that TOR inhibition broadly reduced the transcript abundance of genes involved in auxin and ethylene biosynthesis, transport, and signal transduction (Figure S4). Genetic analysis of hormone mutants and exogenous hormone treatment confirmed that TOR acts downstream or in parallel to auxin and ethylene cascades, and functional TOR activity is essential for hormone-triggered root hair elongation. Meanwhile, sustained TOR signaling maintains basal transcription of hormone-related genes, and disruption of TOR activity results in global downregulation of auxin and ethylene pathway transcripts. Collectively, these genetic and transcriptomic results uncover complex reciprocal interactions between TOR and auxin/ethylene signaling during root hair development, whose detailed regulatory machinery requires further investigation.

RSL4 has previously been characterized as a critical transcriptional hub that integrates auxin and ethylene inputs to regulate root hair growth [22,24]. The data presented in this study show that the expression and accumulation of RSL4 in trichoblasts are affected by glucose–TOR signaling (Figure 4A and Figure 6E,F), positioning TOR upstream of *RSL4* transcription to link carbon nutrient status with root hair growth. The concomitant transcriptional changes in the auxin and ethylene pathways further suggest that these hormonal signals may act parallel to or downstream of TOR to fine-tune root hair development. Root hair development is highly plastic in response to external nutrient and stress conditions, and future work will further elucidate how TOR rewires epidermal transcriptional networks as a central nutrient-sensing switch.

During root hair initiation and elongation in H cells under various conditions, a *bHLH* gene family containing *RHD6* and *RSL4* plays important functions [16,19]. Genetic assays confirmed that glucose cannot trigger root hair growth in the absence of functional RHD6 (Figure 4B–F), and comparative transcriptomics revealed a large set of coexpressed root hair genes that are jointly controlled by TOR and RHD6-RSL1 (Figure 4D). Previous studies demonstrated that the expression of *RHD6* was not regulated by auxin or ethylene [18,22]. Notably, steady-state RHD6 transcript levels are unaffected by TOR inhibition under a continuous supply of sugar (Figure 4A), yet TOR is strictly required for glucose-mediated RHD6 induction following carbon starvation (Figure 4F). This context-dependent transcriptional regulation suggests that TOR alters *RHD6* expression dynamically according to cellular energy status, with additional context-specific regulatory layers remaining to be characterized.

Many nutrient and hormone stimuli trigger root hair elongation via RSL4-dependent transcriptional reprogramming, and *RSL4* is a direct target of RHD6 and other root hair development-related transcription factors, such as ARF5, EIN3 and itself [21,22,24]. It directly binds RHE motifs within RHS gene promoters to activate their expression [21]. The RSL4-RHE regulatory module can drive root hair growth even in the absence of functional RHD6 [19]. Among the 138 annotated RHS genes, 84 are transcriptionally modulated by TOR (77 downregulated, 7 upregulated) (Table S6), confirming TOR as a crucial transcriptional regulator of the root hair effector gene repertoire. The overexpression of either *RHD6* or *RSL4* partially reversed rapamycin-induced root hair defects (Figure 5 and Figure 6), solidifying the role of the RHD6-RSL4 cascade as an essential downstream transcriptional branch of glucose–TOR signaling. Additionally, the current data do not rule out potential posttranslational regulation of RSL4 by TOR signaling. In *Arabidopsis*, TOR is known to modulate protein stability through phosphorylation of downstream substrates [65]. However, the reduced RSL4 protein accumulation upon TOR inhibition observed in this study can be largely explained by transcriptional downregulation (Figure 4 and Figure 6), and the current data cannot establish a direct causal link between TOR activity and RSL4 protein stability. Definitive evidence for posttranslational regulation would require the measurement of RSL4 protein degradation rates in the presence of translation inhibitors, which represents an interesting direction for future investigations.

Extensive prior research has validated the essential role of the *RHD6/RSL* bHLH family in orchestrating root hair morphogenesis [16,19,22,24,57]. Multiple transcription factors converge on this cascade to tune root hair development at the transcriptional level. For example, Feng et al. reported that EIN3 interacted with RHD6 to increase the transcription of RSL4, highlighting that transcription factor complexes shape the transcriptional output of RHD6/RSLs during root hair growth [22]. Bruex et al. reported that more than 1500 genes were differentially expressed in hairy and hairless mutants, indicating that a complex gene regulatory network is involved in the regulation of root hair development [57]. Many transcription factors involved in root hair differentiation, initiation and elongation have been identified in *Arabidopsis* [57]. This study reveals the regulatory relationship between TOR and the RHD6/RSL4 cascade. However, transcriptome data revealed that many genes encoding cellulose synthases, laccases, pectin lyases and xyloglucan endotransglucosylases were downregulated by rapamycin (Table S5), and not all of these genes harbor RHE motifs in their promoter regions, indicating that they may not all be regulated by the RHD6-RSL4 cascade. Some other transcription factors may also be involved in this regulatory network and independent of RHD6/*RSL4* cascades. Transcriptomic data indicate that the expression of 304 transcription factors is regulated by TOR in *Arabidopsis* roots (Figure S4). To predict these potential functions in the regulation of root hair elongation, these cell-specific expression patterns were analyzed on the basis of single-cell RNA sequencing profiles [60]. The results revealed that several TFs were highly preferentially expressed in root hairs and were coexpressed with many root hair-related genes as well as with *RSL4* (Figure S5), suggesting that these TFs, whose functions are unknown, may play a role in root hair development; however, further studies are needed.

This study defines a transcriptional regulatory axis connecting glucose–TOR signaling to root hair morphogenesis. Multiple layers of evidence—including transcriptome profiling, promoter–GUS reporter lines, RT–qPCR quantification, and genetic complementation assays—collectively support that transcriptional reprogramming of the RHD6-RSL4 cascade is a major mechanism underlying TOR-controlled root hair growth. The transcriptomic data presented in this study further revealed that TOR governs the expression of more than 300 root-expressed transcription factors, more than 20 of which are highly enriched in trichoblasts and are tightly coexpressed with *RSL4*. These uncharacterized transcription factors likely act as auxiliary transcriptional coregulators that operate alongside the RHD6-RSL4 cascade to mediate TOR-dependent root hair development, opening clear avenues for future dissection of the full TOR-centered transcriptional network in root epidermal cells.

## 4. Materials and Methods

### 4.1. Plant Materials and Growth Conditions

In this study, the seeds of *Arabidopsis thaliana* ecotype Columbia (Col, used as the wild-type (WT) control for Col-background transgenic lines) and ecotype Wassilewskija (WS, used as the wild-type control for the WS-background *rhd6-1* mutant), along with their derived mutants and transgenic lines, were used for all the experiments. *rhd6-1* (N6347) was obtained from the Nottingham Arabidopsis Stock Centre (NASC) and was described in previous studies [16,72]. All the seeds were sterilized for 5 min in 75% ethanol and then washed with 10% sodium hypochlorite and 0.3% Tween-20 for 7 min. Subsequently, the seeds were washed five times with sterile water. The sterilized seeds were incubated at 4 °C in the dark for 2 days. Finally, the sterilized seeds or seedlings were grown on a 0.5× Murashige and Skoog (MS) medium in growth chambers at 22 °C under a 16 h light/8 h dark photoperiod. The 0.5× MS medium contained 825.0 mg/L ammonium nitrate, 3.1 mg/L boric acid, 166.1 mg/L anhydrous calcium chloride, 0.0125 mg/L cobalt chloride·6H_2_O, 0.0125 mg/L cupric sulfate·5H_2_O, 18.63 mg/L Na_2_EDTA·2H_2_O, 13.9 mg/L ferrous sulfate·7H_2_O, 90.35 mg/L anhydrous magnesium sulfate, 8.45 mg/L manganese sulfate·H_2_O, 0.125 mg/L sodium molybdate·2H_2_O, 0.415 mg/L potassium iodide, 950.0 mg/L potassium nitrate, 85.0 mg/L monobasic potassium phosphate, 4.3 mg/L zinc sulfate·7H_2_O; organic components: 1.0 mg/L glycine, 50.0 mg/L myo-inositol, 0.25 mg/L nicotinic acid, 0.25 mg/L pyridoxine·HCl, 0.05 mg/L thiamine·HCl. Plant agar was supplemented at 7 g/L. For standard growth medium, 10 g/L sucrose was added. Sucrose was omitted for sugar-free culture assays. The pH was adjusted to 5.7–5.8 with 1 M KOH prior to autoclaving at 121 °C for 20 min. Filter-sterilized D-glucose, rapamycin, AZD8055 and MG132 were added to autoclaved medium after cooling to 50–60 °C.

### 4.2. Cloning of RHD6 and RSL4 and Transformation into BP12-2

The full-length coding sequences of *RHD6* (897 bp) and *RSL4* (777 bp) were amplified from cDNA templates prepared from wild-type *Arabidopsis* seedlings. The amplified products were subsequently cloned and inserted into P35S::8GWN, a gateway system-based entry vector, to generate the recombinant plasmids. These recombinant constructs were subsequently transformed into pEarleyGate303 through a Gateway recombination system [35]. Finally, the constructed vectors were introduced into *Agrobacterium tumefaciens* strain *GV3101* and used in the transformation of BP12-2 (a yeast FKBP12 overexpression line) [35]. The floral dip method was used in the transformation of *Arabidopsis* [73]. Positive transgenic lines were selected on a 0.5 MS medium supplemented with 50 mg/L kanamycin.

### 4.3. Generation of EXPA7 Promoter–GUS Fusion Plants and RSL4pro::RSL4-GFP Plants

The promoter sequence of *EXPA7* (725 bp) was amplified from the genomic DNA of WT *Arabidopsis*. The amplified product was cloned and inserted into Pro35S::8GWN to replace the *Cauliflower mosaic virus* 35S promoter and was subsequently fused with the *GUS* gene. These recombinant constructs were subsequently transformed into pEarleyGate303 to generate *EXPA7* promoter–GUS fusion plants. For the *RSL4pro::RSL4-GFP* plants, the promoter sequence of *RSL4* (2455 bp) was amplified from the genomic DNA of WT *Arabidopsis*, and the *RSL4* gene (777 bp) was fused with the *green fluorescent protein* (*GFP*) gene. The *RSL4* promoter fragment and *RSL4-GFP* fragment were introduced into p8GWN to generate an *RSL4pro::RSL4-GFP* vector for transformation of *Arabidopsis*.

### 4.4. Root Hair Length Measurement

In this study, the root hair of the root maturation zones or the root tips of the seedlings was photographed with a stereomicroscope, and the length of the root hair was measured by using the software ImageJ software (v1.8.0). Two root hairs were measured from each of 10 independent seedlings per biological replicate, and individual seedlings represented the experimental unit. Multiple root-hair measurements from a single seedling were treated as subsamples. Three independent biological replicates were performed.

### 4.5. GUS Staining

Histochemical GUS staining was performed as previously described with minor modifications [64]. The 5-DAG seedlings with corresponding treatments were immersed in GUS staining buffer containing 50 mM sodium phosphate buffer (pH 7.0), 10 mM EDTA, 0.5 mM potassium ferricyanide, 0.5 mM potassium ferrocyanide, 0.1% (*v*/*v*) Triton X-100, and 1 mM X-Gluc (5-bromo-4-chloro-3-indolyl-β-D-glucuronide cyclohexylammonium salt), followed by incubation at 37 °C in the dark. After sufficient color development, seedlings were cleared with 70% ethanol to remove chlorophyll, and the stained root tissues were observed under a stereomicroscope.

### 4.6. Confocal Microscopy and GFP Fluorescence Quantification

Root tissues of RSL4pro::RSL4-GFP transgenic seedlings were mounted in 10 mM phosphate-buffered saline for confocal observation. GFP fluorescence signals were captured using a laser-scanning confocal microscope. GFP excitation wavelength was set to 488 nm, and emission signal was collected within the 500–530 nm range. Identical laser power, gain, offset and pinhole parameters were strictly maintained across all sample groups to guarantee comparable fluorescence intensity. For quantitative analysis of nuclear RSL4-GFP fluorescence intensity in trichoblast cells, raw confocal images were processed using ImageJ software (v1.8.0). Nuclei of trichoblast cells were manually outlined as regions of interest (ROIs). Mean gray-value fluorescence intensity was measured for each selected nucleus, and background fluorescence from adjacent cell-free regions was subtracted from each ROI value. Background-corrected fluorescence values were normalized to the mean value of the DMSO control group, which was set to 1, to calculate relative fluorescence intensity for cross-group comparisons.

### 4.7. RNA Extraction and Real-Time Quantitative PCR (RT–qPCR)

Total RNA was extracted from different samples using the RNAprep Pure Plant Kit (TianGen Biotech, Beijing, China) and was detected on 1% agarose gels. Approximately 1 μg of total RNA from each sample was used for reverse transcription. First-strand cDNA was synthesized by the PrimeScript RT Reagent Kit (TaKaRa, Kusatsu, Shiga, Japan). The cDNAs were used as templates for the RT–qPCR. The RT–qPCR was conducted using a SYBR Premix Ex Taq II kit (TaKaRa, Kusatsu, Shiga, Japan) on a LightCycler480 II system (Roche Diagnostics, Mannheim, Baden-Württemberg, Germany). RT–qPCR was performed in a volume of 20 μL using the following conditions: 94 °C predenaturation for 1 min, 40 cycles of 94 °C for 5 s, and 60 °C for 30 s. Three independent biological replicates were performed, and each biological replicate consists of tissue pooled from multiple independent *Arabidopsis* seedlings, with three technical replicate RT-qPCR reactions assayed per pooled cDNA sample. *Actin2* was used as an internal reference gene, and the relative expression of genes was calculated by using the 2^−ΔΔc(t)^ method. The primers used for RT–qPCR are listed in Table S9.

### 4.8. Transcriptome Sequencing and Data Analysis

BP12-2 seedlings were grown vertically on 0.5 MS medium for 6 d and then treated with rapamycin (5 µM) or DMSO for 24 h or 48 h for transcriptome sequencing sample collecting. Three biological replicates were prepared for each condition. Root total RNA was extracted using the RNAprep Pure Plant Kit (TianGen Biotech). RNA quality was evaluated by a NanoPhotometer spectrophotometer (IMPLEN, Munich, Bavaria, Germany) and 2100 Bioanalyzer system (Agilent Technologies, Santa Clara, CA, USA). The NEBNext Ultra^TM^ RNA Library Prep Kit for Illumina (New England Biolabs, Ipswich, MA, USA) was used to prepare sequencing libraries following the manufacturer’s recommendations. Deep sequencing was performed on an Illumina HiSeq 2000 platform (Illumina, San Diego, CA, USA), and 150 bp paired-end reads were generated. Adapter sequences and low-quality reads were removed from the raw reads, and high-quality clean reads were aligned to the reference genome (TAIR10, https://www.arabidopsis.org (accessed on 15 March 2025)). The fragments per kilobase of transcript per million mapped reads (FPKM) of each gene were calculated on the basis of the length of the gene and the read count mapped to this gene. The DESeq2 software package (v1.42.0) was used for differential gene expression analysis [74]. Genes with an adjusted *p* value < 0.05 according to DESeq2 were considered differentially expressed genes. Raw sequencing data have been deposited in the National Genomics Data Center (BioProject PRJCA071398 and Genome Sequence Archive (GSA) accession CRA048082).

For the genes that were differentially expressed between the *rhd6-3 rsl1-1* and WT strains used in this study, data were downloaded from the GEO database with accession no. GSE107699, and the genes that were differentially expressed were analyzed by the edgeR software package (v4.2.1) [22,75]. The RHD6/RSL1-regulated genes were identified using the following cutoff values: expression FC greater than or equal to 2 and an FDR less than 0.01.

## 5. Conclusions

In summary, multilayered transcriptomic and genetic analyses establish a clear molecular model for TOR-mediated root hair elongation in *Arabidopsis*. Glucose-activated TOR kinase acts as an upstream nutrient sensor that drives root hair growth predominantly through transcriptional regulation of the RHD6-RSL4 bHLH cascade. TOR maintains robust transcription of RHD6 and RSL4, and elevated RSL4 levels drive the expression of hundreds of *RHS* genes encoding cell wall remodeling and the ROS metabolic machinery required for polar root hair outgrowth. Loss of TOR activity strongly represses *RSL1*–*RSL5* transcription, abolishes glucose- and hormone-triggered root hair elongation, and disrupts the full epidermal transcriptional network. Genetic overexpression of RHD6 or RSL4 partially bypasses TOR inhibition to restore root hair growth, confirming their position as core downstream transcriptional effectors of TOR. Beyond the RHD6-RSL4 module, TOR regulates more than 20 uncharacterized trichoblast-enriched transcription factors that function in parallel to control epidermal development. This work establishes a transcriptional framework linking carbon nutrient signaling to root morphogenesis, demonstrating that the RHD6-RSL4 cascade acts as a core downstream transcriptional module of glucose–TOR signaling in root hair elongation. These findings provide a foundation for future investigations of both transcriptional and potential posttranslational mechanisms underlying TOR-mediated root development.

## Data Availability

The RNA-seq raw data generated in this study have been deposited in the National Genomics Data Center (BioProject PRJCA071398 and GSA accession CRA048082). All other supporting data are available from the corresponding author upon reasonable request.

## Supplementary Materials

The following supporting information can be downloaded at: https://www.mdpi.com/article/10.3390/plants15172586/s1, Figure S1: Auxin and ethylene require functional TOR signaling to promote root hair elongation in *Arabidopsis*; Figure S2: Genetic interaction between TOR and auxin/ethylene signaling pathways regulates root hair elongation; Figure S3: Overview of DEGs in rapamycin-treated seedling roots compared with DMSO control; Figure S4: Differentially expressed genes involved in auxin and ethylene biosynthesis and signal transduction after TOR inhibition; Figure S5: Summary of transcription factors with altered expression after rapamycin treatment in *Arabidopsis* seedling roots; Figure S6: Co-expression overlap between rapamycin-repressed genes and trichoblast-enriched transcription factor target genes; Figure S7: RT–qPCR quantification of representative RHS gene transcript levels in WT roots treated with 1 μM AZD8055 or DMSO for 24 h; Figure S8: TOR inhibitors repress the accumulation of the RSL4 protein in trichoblasts; Figure S9: Quantification of RSL4-GFP fluorescence intensity corresponding to Figure 6E,F; Table S1: Differentially expressed genes regulated by rapamycin; Table S2: TOR-regulated genes among 138 root hair development-related genes in *Arabidopsis*; Table S3: List of the overlapping genes between genes identified in this study and the published gene set from Angela Bruex et al. [57]; Table S4: GO enrichment analysis of the overlapping genes between genes identified in this study and the published gene set from Angela Bruex et al.; Table S5: TOR-regulated cell wall-related genes and peroxidase; Table S6: List of the overlapping genes between genes identified in this study and putative RSL4 target genes containing the RHE in Hwang et al. [21]; Table S7: Differentially expressed genes in *rhd6 rsl1*; Table S8: GO enrichment of TOR and RHD6/RSL1 co-regulated genes; Table S9: Primers used in this study.

## References

[1] Grierson C., Schiefelbein J. (2002). Root hairs. Arab. Book.

[2] Ishida T., Kurata T., Okada K., Wada T. (2008). A genetic regulatory network in the development of trichomes and root hairs. Annu. Rev. Plant Biol..

[3] Salazar-Henao J.E., Velez-Bermudez I.C., Schmidt W. (2016). The regulation and plasticity of root hair patterning and morphogenesis. Development.

[4] Cui S., Suzaki T., Tominaga-Wada R., Yoshida S. (2018). Regulation and functional diversification of root hairs. Semin. Cell Dev. Biol..

[5] Masucci J.D., Rerie W.G., Foreman D.R., Zhang M., Galway M.E., Marks M.D., Schiefelbein J.W. (1996). The homeobox gene GLABRA2 is required for position-dependent cell differentiation in the root epidermis of *Arabidopsis thaliana*. Development.

[6] Schiefelbein J., Kwak S.H., Wieckowski Y., Barron C., Bruex A. (2009). The gene regulatory network for root epidermal cell-type pattern formation in *Arabidopsis*. J. Exp. Bot..

[7] Lee M.M., Schiefelbein J. (1999). WEREWOLF, a MYB-related protein in *Arabidopsis*, is a position-dependent regulator of epidermal cell patterning. Cell.

[8] Galway M.E., Masucci J.D., Lloyd A.M., Walbot V., Davis R.W., Schiefelbein J.W. (1994). The TTG gene is required to specify epidermal cell fate and cell patterning in the *Arabidopsis* root. Dev. Biol..

[9] Bernhardt C., Lee M.M., Gonzalez A., Zhang F., Lloyd A., Schiefelbein J. (2003). The bHLH genes *GLABRA3 (GL3)* and *ENHANCER OF GLABRA3 (EGL3)* specify epidermal cell fate in the *Arabidopsis* root. Development.

[10] Zhang F., Gonzalez A., Zhao M., Payne C.T., Lloyd A. (2003). A network of redundant bHLH proteins functions in all TTG1-dependent pathways of *Arabidopsis*. Development.

[11] Kurata T., Ishida T., Kawabata-Awai C., Noguchi M., Hattori S., Sano R., Nagasaka R., Tominaga R., Koshino-Kimura Y., Kato T. (2005). Cell-to-cell movement of the CAPRICE protein in *Arabidopsis* root epidermal cell differentiation. Development.

[12] Song S.K., Ryu K.H., Kang Y.H., Song J.H., Cho Y.H., Yoo S.D., Schiefelbein J., Lee M.M. (2011). Cell fate in the *Arabidopsis* root epidermis is determined by competition between WEREWOLF and CAPRICE. Plant Physiol..

[13] Bernhardt C., Zhao M., Gonzalez A., Lloyd A., Schiefelbein J. (2005). The bHLH genes GL3 and EGL3 participate in an intercellular regulatory circuit that controls cell patterning in the *Arabidopsis* root epidermis. Development.

[14] Cheng Y., Zhu W., Chen Y., Ito S., Asami T., Wang X. (2014). Brassinosteroids control root epidermal cell fate via direct regulation of a MYB-bHLH-WD40 complex by GSK3-like kinases. eLife.

[15] Masucci J.D., Schiefelbein J.W. (1996). Hormones act downstream of TTG and GL2 to promote root hair outgrowth during epidermis development in the *Arabidopsis* root. Plant Cell.

[16] Masucci J.D., Schiefelbein J.W. (1994). The rhd6 Mutation of *Arabidopsis thaliana* Alters Root-Hair Initiation through an Auxin- and Ethylene-Associated Process. Plant Physiol..

[17] Menand B., Yi K., Jouannic S., Hoffmann L., Ryan E., Linstead P., Schaefer D.G., Dolan L. (2007). An ancient mechanism controls the development of cells with a rooting function in land plants. Science.

[18] Pires N.D., Yi K., Breuninger H., Catarino B., Menand B., Dolan L. (2013). Recruitment and remodeling of an ancient gene regulatory network during land plant evolution. Proc. Natl. Acad. Sci. USA.

[19] Yi K., Menand B., Bell E., Dolan L. (2010). A basic helix-loop-helix transcription factor controls cell growth and size in root hairs. Nat. Genet..

[20] Han X., Zhang M., Yang M., Hu Y. (2020). *Arabidopsis* JAZ Proteins Interact with and Suppress RHD6 Transcription Factor to Regulate Jasmonate-Stimulated Root Hair Development. Plant Cell.

[21] Hwang Y., Choi H.-S., Cho H.-M., Cho H.-T. (2017). Tracheophytes Contain Conserved Orthologs of a Basic Helix-Loop-Helix Transcription Factor That Modulate *ROOT HAIR SPECIFIC* Genes. Plant Cell.

[22] Feng Y., Xu P., Li B., Li P., Wen X., An F., Gong Y., Xin Y., Zhu Z., Wang Y. (2017). Ethylene promotes root hair growth through coordinated EIN3/EIL1 and RHD6/RSL1 activity in *Arabidopsis*. Proc. Natl. Acad. Sci. USA.

[23] Vijayakumar P., Datta S., Dolan L. (2016). ROOT HAIR DEFECTIVE SIX-LIKE4 (RSL4) promotes root hair elongation by transcriptionally regulating the expression of genes required for cell growth. New Phytol..

[24] Mangano S., Denita-Juarez S.P., Choi H.-S., Marzol E., Hwang Y., Ranocha P., Velasquez S.M., Borassi C., Barberini M.L., Aptekmann A.A. (2017). Molecular link between auxin and ROS-mediated polar growth. Proc. Natl. Acad. Sci. USA.

[25] Shi L., Wu Y., Sheen J. (2018). TOR signaling in plants: Conservation and innovation. Development.

[26] Sehgal S.N., Baker H., Vezina C. (1975). Rapamycin (AY-22,989), a new antifungal antibiotic. II. Fermentation, isolation and characterization. J. Antibiot..

[27] Kunz J., Henriquez R., Schneider U., Deuter-Reinhard M., Movva N.R., Hall M.N. (1993). Target of rapamycin in yeast, TOR2, is an essential phosphatidylinositol kinase homolog required for G1 progression. Cell.

[28] Sabatini D.M., Erdjument-Bromage H., Lui M., Tempst P., Snyder S.H. (1994). RAFT1: A mammalian protein that binds to FKBP12 in a rapamycin-dependent fashion and is homologous to yeast TORs. Cell.

[29] Laplante M., Sabatini D.M. (2012). mTOR signaling in growth control and disease. Cell.

[30] Menand B., Desnos T., Nussaume L., Berger F., Bouchez D., Meyer C., Robaglia C. (2002). Expression and disruption of the *Arabidopsis* TOR (target of rapamycin) gene. Proc. Natl. Acad. Sci. USA.

[31] Xiong Y., Sheen J. (2014). The Role of Target of Rapamycin Signaling Networks in Plant Growth and Metabolism. Plant Physiol..

[32] Xu Q., Liang S., Kudla J., Luan S. (1998). Molecular characterization of a plant FKBP12 that does not mediate action of FK506 and rapamycin. Plant J..

[33] Deprost D., Yao L., Sormani R., Moreau M., Leterreux G., Nicolai M., Bedu M., Robaglia C., Meyer C. (2007). The *Arabidopsis* TOR kinase links plant growth, yield, stress resistance and mRNA translation. EMBO Rep..

[34] Xiong Y., McCormack M., Li L., Hall Q., Xiang C., Sheen J. (2013). Glucose-TOR signalling reprograms the transcriptome and activates meristems. Nature.

[35] Ren M., Venglat P., Qiu S., Feng L., Cao Y., Wang E., Xiang D., Wang J., Alexander D., Chalivendra S. (2012). Target of rapamycin signaling regulates metabolism, growth, and life span in *Arabidopsis*. Plant Cell.

[36] Sormani R., Yao L., Menand B., Ennar N., Lecampion C., Meyer C., Robaglia C. (2007). Saccharomyces cerevisiae FKBP12 binds *Arabidopsis thaliana* TOR and its expression in plants leads to rapamycin susceptibility. BMC Plant Biol..

[37] Caldana C., Li Y., Leisse A., Zhang Y., Bartholomaeus L., Fernie A.R., Willmitzer L., Giavalisco P. (2013). Systemic analysis of inducible target of rapamycin mutants reveal a general metabolic switch controlling growth in *Arabidopsis thaliana*. Plant J..

[38] Montané M.-H., Menand B. (2013). ATP-competitive mTOR kinase inhibitors delay plant growth by triggering early differentiation of meristematic cells but no developmental patterning change. J. Exp. Bot..

[39] Xiong F., Zhang R., Meng Z., Deng K., Que Y., Zhuo F., Feng L., Guo S., Datla R., Ren M. (2017). Brassinosteriod Insensitive 2 (BIN2) acts as a downstream effector of the Target of Rapamycin (TOR) signaling pathway to regulate photoautotrophic growth in *Arabidopsis*. New Phytol..

[40] Deng K., Dong P., Wang W., Feng L., Xiong F., Wang K., Zhang S., Feng S., Wang B., Zhang J. (2017). The TOR Pathway Is Involved in Adventitious Root Formation in *Arabidopsis* and Potato. Front. Plant Sci..

[41] Van Leene J., Han C., Gadeyne A., Eeckhout D., Matthijs C., Cannoot B., De Winne N., Persiau G., Van De Slijke E., Van de Cotte B. (2019). Capturing the phosphorylation and protein interaction landscape of the plant TOR kinase. Nat. Plants.

[42] Dobrenel T., Caldana C., Hanson J., Robaglia C., Vincentz M., Veit B., Meyer C. (2016). TOR Signaling and Nutrient Sensing. Annu. Rev. Plant Biol..

[43] Wang P., Zhao Y., Li Z., Hsu C.C., Liu X., Fu L., Hou Y.J., Du Y., Xie S., Zhang C. (2018). Reciprocal Regulation of the TOR Kinase and ABA Receptor Balances Plant Growth and Stress Response. Mol. Cell.

[44] Li X., Cai W., Liu Y., Li H., Fu L., Liu Z., Xu L., Liu H., Xu T., Xiong Y. (2017). Differential TOR activation and cell proliferation in *Arabidopsis* root and shoot apexes. Proc. Natl. Acad. Sci. USA.

[45] Schepetilnikov M., Makarian J., Srour O., Geldreich A., Yang Z., Chicher J., Hammann P., Ryabova L.A. (2017). GTPase ROP2 binds and promotes activation of target of rapamycin, TOR, in response to auxin. EMBO J..

[46] Ahn C.S., Han J.A., Lee H.S., Lee S., Pai H.S. (2011). The PP2A regulatory subunit Tap46, a component of the TOR signaling pathway, modulates growth and metabolism in plants. Plant Cell.

[47] Mahfouz M.M., Kim S., Delauney A.J., Verma D.P. (2006). *Arabidopsis* TARGET OF RAPAMYCIN interacts with RAPTOR, which regulates the activity of S6 kinase in response to osmotic stress signals. Plant Cell.

[48] Forzani C., Duarte G.T., Van Leene J., Clement G., Huguet S., Paysant-Le-Roux C., Mercier R., De Jaeger G., Leprince A.S., Meyer C. (2019). Mutations of the AtYAK1 Kinase Suppress TOR Deficiency in *Arabidopsis*. Cell Rep..

[49] Dobrenel T., Mancera-Martinez E., Forzani C., Azzopardi M., Davanture M., Moreau M., Schepetilnikov M., Chicher J., Langella O., Zivy M. (2016). The *Arabidopsis* TOR Kinase Specifically Regulates the Expression of Nuclear Genes Coding for Plastidic Ribosomal Proteins and the Phosphorylation of the Cytosolic Ribosomal Protein S6. Front. Plant Sci..

[50] Ahn C.S., Ahn H.K., Pai H.S. (2015). Overexpression of the PP2A regulatory subunit Tap46 leads to enhanced plant growth through stimulation of the TOR signalling pathway. J. Exp. Bot..

[51] Zhang Z., Zhu J.Y., Roh J., Marchive C., Kim S.K., Meyer C., Sun Y., Wang W., Wang Z.Y. (2016). TOR Signaling Promotes Accumulation of BZR1 to Balance Growth with Carbon Availability in *Arabidopsis*. Curr. Biol..

[52] Xiong Y., Sheen J. (2012). Rapamycin and glucose-target of rapamycin (TOR) protein signaling in plants. J. Biol. Chem..

[53] Leiber R.M., John F., Verhertbruggen Y., Diet A., Knox J.P., Ringli C. (2010). The TOR pathway modulates the structure of cell walls in *Arabidopsis*. Plant Cell.

[54] Monreal Contreras H.A., Arthikala M.-K., Lara M., Nanjareddy K. (2025). Target of Rapamycin is involved in root hair development in Phaseolus vulgaris. Plant Signal. Behav..

[55] Pitts R.J., Cernac A., Estelle M. (1998). Auxin and ethylene promote root hair elongation in *Arabidopsis*. Plant J..

[56] Kwasniewski M., Nowakowska U., Szumera J., Chwialkowska K., Szarejko I. (2013). iRootHair: A comprehensive root hair genomics database. Plant Physiol..

[57] Bruex A., Kainkaryam R.M., Wieckowski Y., Kang Y.H., Bernhardt C., Xia Y., Zheng X., Wang J.Y., Lee M.M., Benfey P. (2012). A gene regulatory network for root epidermis cell differentiation in *Arabidopsis*. PLoS Genet..

[58] Baumberger N., Ringli C., Keller B. (2001). The chimeric leucine-rich repeat/extensin cell wall protein LRX1 is required for root hair morphogenesis in *Arabidopsis thaliana*. Genes Dev..

[59] Foreman J., Demidchik V., Bothwell J.H., Mylona P., Miedema H., Torres M.A., Linstead P., Costa S., Brownlee C., Jones J.D. (2003). Reactive oxygen species produced by NADPH oxidase regulate plant cell growth. Nature.

[60] Zhang T.Q., Xu Z.G., Shang G.D., Wang J.W. (2019). A Single-Cell RNA Sequencing Profiles the Developmental Landscape of *Arabidopsis* Root. Mol. Plant.

[61] Kim D.W., Lee S.H., Choi S.B., Won S.K., Heo Y.K., Cho M., Park Y.I., Cho H.T. (2006). Functional conservation of a root hair cell-specific cis-element in angiosperms with different root hair distribution patterns. Plant Cell.

[62] Cho H.T., Cosgrove D.J. (2002). Regulation of root hair initiation and expansin gene expression in *Arabidopsis*. Plant Cell.

[63] Schepetilnikov M., Dimitrova M., Mancera-Martínez E., Geldreich A., Keller M., Ryabova L.A. (2013). TOR and S6K1 promote translation reinitiation of uORF-containing mRNAs via phosphorylation of eIF3h. EMBO J..

[64] Deng K., Yu L., Zheng X., Zhang K., Wang W., Dong P., Zhang J., Ren M. (2016). Target of Rapamycin Is a Key Player for Auxin Signaling Transduction in *Arabidopsis*. Front. Plant Sci..

[65] Yuan X., Xu P., Yu Y., Xiong Y. (2020). Glucose-TOR signaling regulates PIN2 stability to orchestrate auxin gradient and cell expansion in *Arabidopsis* root. Proc. Natl. Acad. Sci. USA.

[66] Retzer K., Weckwerth W. (2021). The TOR-Auxin Connection Upstream of Root Hair Growth. Plants.

[67] Zhuo F., Xiong F., Deng K., Li Z., Ren M. (2020). Target of Rapamycin (TOR) Negatively Regulates Ethylene Signals in *Arabidopsis*. Int. J. Mol. Sci..

[68] Fu L., Liu Y., Qin G., Wu P., Zi H., Xu Z., Zhao X., Wang Y., Li Y., Yang S. (2021). The TOR–EIN2 axis mediates nuclear signalling to modulate plant growth. Nature.

[69] Bahamonde C., de la Osa C., Rexach J., Herrera-Rodríguez M.B., Gabarain V.B., Estevez J.M., Navarro-Gochicoa M.T., González-Fontes A., Camacho-Cristóbal J.J. (2026). Auxin-Dependent Activation of RHD6-RSL4 Cascade Promotes Root Hair Growth Under Boron Deficiency in *Arabidopsis* Primary Root Apices. Plant Cell Environ..

[70] Villaécija-Aguilar J.A., Körösy C., Maisch L., Hamon-Josse M., Petrich A., Magosch S., Chapman P., Bennett T., Gutjahr C. (2022). KAI2 promotes *Arabidopsis* root hair elongation at low external phosphate by controlling local accumulation of AUX1 and PIN2. Curr. Biol..

[71] Song L., Yu H., Dong J., Che X., Jiao Y., Liu D. (2016). The Molecular Mechanism of Ethylene-Mediated Root Hair Development Induced by Phosphate Starvation. PLoS Genet..

[72] Deruere J., Jackson K., Garbers C., Soll D., Delong A. (1999). The RCN1-encoded A subunit of protein phosphatase 2A increases phosphatase activity in vivo. Plant J..

[73] Zhang X., Henriques R., Lin S.S., Niu Q.W., Chua N.H. (2006). Agrobacterium-mediated transformation of *Arabidopsis thaliana* using the floral dip method. Nat. Protoc..

[74] Love M.I., Huber W., Anders S. (2014). Moderated estimation of fold change and dispersion for RNA-seq data with DESeq2. Genome Biol..

[75] Robinson M.D., McCarthy D.J., Smyth G.K. (2010). edgeR: A Bioconductor package for differential expression analysis of digital gene expression data. Bioinformatics.

